# Health Care Usage During the COVID-19 Pandemic and the Adoption of Telemedicine: Retrospective Study of Chronic Disease Cohorts

**DOI:** 10.2196/54991

**Published:** 2024-10-03

**Authors:** Margrét Vilborg Bjarnadóttir, David Anderson, Kelley M Anderson, Omar Aljwfi, Alina Peluso, Adam Ghannoum, Gayle Balba, Nawar Shara

**Affiliations:** 1 Decisions, Operations and Information Technology University of Maryland, College Park College Park, MD United States; 2 Villanova School of Business Villanova, PA United States; 3 School of Nursing Georgetown University Washington, DC United States; 4 MedStar Health Research Institute Hyattsville, MD United States; 5 Oak Ridge National Laboratory Oak Ridge, TN United States; 6 University of Maryland, College Park College Park, MD United States; 7 Department of Infectious Diseases MedStar Georgetown University Hospital Washington, DC United States; 8 Department of Endocrinology MedStar Georgetown University Hospital Washington, DC United States

**Keywords:** telehealth utilization, health care utilization, demographic differences, cohort study, telehealth, COVID-19, telehealth adaption

## Abstract

**Background:**

The COVID-19 pandemic accelerated telehealth adoption across disease cohorts of patients. For many patients, routine medical care was no longer an option, and others chose not to visit medical offices in order to minimize COVID-19 exposure. In this study, we take a comprehensive multidisease approach in studying the impact of the COVID-19 pandemic on health care usage and the adoption of telemedicine through the first 12 months of the COVID-19 pandemic.

**Objective:**

We studied the impact of the COVID-19 pandemic on in-person health care usage and telehealth adoption across chronic diseases to understand differences in telehealth adoption across disease cohorts and patient demographics (such as the Social Vulnerability Index [SVI]).

**Methods:**

We conducted a retrospective cohort study of 6 different disease cohorts (anxiety: n=67,578; depression: n=45,570; diabetes: n=81,885; kidney failure: n=29,284; heart failure: n=21,152; and cancer: n=35,460). We used summary statistics to characterize changes in usage and regression analysis to study how patient characteristics relate to in-person health care and telehealth adoption and usage during the first 12 months of the pandemic.

**Results:**

We observed a reduction in in-person health care usage across disease cohorts (ranging from 10% to 24%). For most diseases we study, telehealth appointments offset the reduction in in-person visits. Furthermore, for anxiety and depression, the increase in telehealth usage exceeds the reduction in in-person visits (by up to 5%). We observed that younger patients and men have higher telehealth usage after accounting for other covariates. Patients from higher SVI areas are less likely to use telehealth; however, if they do, they have a higher number of telehealth visits, after accounting for other covariates.

**Conclusions:**

The COVID-19 pandemic affected health care usage across diseases, and the role of telehealth in replacing in-person visits varies by disease cohort. Understanding these differences can inform current practices and provides opportunities to further guide modalities of in-person and telehealth visits. Critically, further study is needed to understand barriers to telehealth service usage for patients in higher SVI areas. A better understanding of the role of social determinants of health may lead to more support for patients and help individual health care providers improve access to care for patients with chronic conditions.

## Introduction

The COVID-19 pandemic exerted both direct and indirect impacts on individual health and well-being. Prior to March 2020, routine clinic visits were performed as preventative measures to identify conditions that may result in severe diseases and as a component of chronic condition management. When COVID-19 lockdowns were instituted across the United States, many clinical offices and laboratories closed. Routine medical care was no longer an option for many patients, and in other cases patients chose not to visit medical offices in order to minimize COVID-19 exposure.

Across studies, the median reduction in health care usage during the pandemic (across visits, admissions, diagnostics, and therapeutics) was 37%, with healthier individuals being more likely to reduce their use of health care services [1]. Many patients with chronic disease experienced detrimental effects: the literature documents a significant decrease in the treatment and detection of chronic disease [2,3], as well as adverse effects on chronic disease management as patients reduced their usage and experienced barriers in access to care. For example, New York City witnessed a 139% increase in deaths related to heart disease during the pandemic [4]. Experts attribute this increase in mortality to a decrease in diagnostic procedures as patients were unable to access facilities and as those facilities experienced an overwhelming influx of patients with COVID-19. Cancer rates also increased during the pandemic. For instance, due to COVID-19 lockdowns, routine breast cancer screening was suspended throughout 2020, and it is anticipated that this delay in detection will lead to an estimated 7.9%-9.6% short-term increase in deaths due to breast cancer [5].

Beyond the numerous studies that have evaluated health care outcomes, there is a growing body of studies about health care resource usage during and post the pandemic [6-8] and about the widespread adoption of telehealth. Telehealth, that is, the use of audio and video consultations, is time-efficient, socially distanced, and low cost [9]. Therefore, unsurprisingly, the use of telehealth surged during the pandemic, as many of the barriers to its adoption were removed to support clinical care [10]. Telehealth was used by patients who were immunocompromised and by others wary of possible COVID-19 infection or affected by lockdowns [9].

While telemedicine consultations are not suitable for all patients, the pandemic has permanently changed telemedicine usage patterns, and the use of telemedicine may well become a new standard of care, although patient and health care providers’ satisfaction and views are mixed [11-16]. Therefore, disparities in the adoption of telehealth are an important consideration. Such disparities have been documented in a number of studies, which show that overall, there is less adoption among older patients [17,18], patients of lower socioeconomic status [17-21], minorities [18,20,21] and men [19,20]. These disparities are driven by lack of access to the necessary technology, particularly for patients of lower socioeconomic status and older patients [19].

In this study, we take a comprehensive approach to study health care usage and the adoption of telemedicine through the first 12 months of the COVID-19 pandemic. We selected conditions that are common with high health care usage in the categories of mental health (depression and anxiety), cardiovascular disease (heart failure), cancer, renal disease (kidney failure), and endocrine disorder (diabetes) [22-24]. We also evaluated the impact of specific social determinants of health indicators on health care usage in general and telehealth adoption in particular. Importantly, this study allows us to compare the usage and adoption of telehealth across chronic disease cohorts in the context of health care disparities. We aim to contribute to our understanding of how various patient populations used telemedicine during the pandemic, which can inform improved access to care for vulnerable groups.

## Methods

### Cohort Definition

Information about diagnosis and usage was extracted from the electronic health records of a regional health care system serving Virginia, Maryland, and the District of Columbia. For each of our study diseases (anxiety, depression, diabetes, kidney failure, heart failure, and cancer), we defined a disease cohort of patients to include any patient diagnosed with the disease before October 2019. The corresponding *ICD-9* (*International Classification of Diseases, Ninth Revision*) and *ICD-10* (*International Statistical Classification of Diseases, Tenth Revision*) codes are provided in Section A of Multimedia Appendix 1. Since a single patient experiencing multiple chronic diseases may be in more than 1 disease cohort, we conducted a robustness check that independently repeated the analysis only on those members who are in a single disease cohort. The results are materially the same and are included in Section B of Multimedia Appendix 1.

We then evaluated members’ health care usage in 6 months prior to the pandemic lockdowns, from October 2019 through March 2020. We next compared usage in this period with usage during a 12-month pandemic period, from June 2020 through May 2021. Note that we excluded April and May 2020 from the study, as health care services in the system under study were significantly disrupted during that period.

### Demographic, Socioeconomic, and Clinical Variables

We extracted each patient’s age, gender, race, and county from the electronic health records data. For gender, due to low numbers in the nonbinary and not reported categories, we grouped all patients in those categories together. Similarly, and also due to low numbers, we grouped together all patients not identified as Black or White. We extracted the Federal Information Processing Standards county-level Social Vulnerability Index (SVI) from the Centers for Disease Control and Prevention [25] and matched it with the patient’s zip code. Finally, we calculated each member’s Charlson Comorbidity Index, based on the patient’s visits from January 2018 to the end of the prepandemic period. As we are explicitly using age in our models, the age-free version was calculated [26].

### Usage

Before the pandemic telehealth services were available, primarily through an eVisit app, telehealth services were available across various ambulatory sites, offering options such as video visits, audio-only phone visits, and eConsults. Despite the availability, usage remained modest. With the pandemic onset there was a significant surge in telehealth demand, peaking at more than 400 visits daily. In response, strategic adaptations were made, including temporarily waiving patient fees until June 30, 2021. In this study, we define a telehealth visit as both video and audio visits. We defined in-person usage as the number of inpatient, outpatient, and emergency department visits in a given period for any reason (not disease-specific). We then defined overall usage as the sum of in-person usage and the number of telehealth visits.

### Statistical Analysis

We conducted a longitudinal cohort study comparing usage using summary statistics and regression analysis. First, for each disease we compared the in-person and overall usage in the prepandemic period with usage during the pandemic time period (broken into two 6-month subperiods to highlight temporal patterns).

Then, for each disease, we constructed a regression model that explains the number of pandemic period in-person visits as a function of the usage in the prepandemic period and patients’ demographic, socioeconomic, and clinical characteristics. Because the distribution of in-person visits is left-skewed, we transformed the dependent variable to ensure statistical fit. Specifically, we fit a regression model with the natural logarithm of the pandemic period in-person visits as the dependent variable and in-person visits in the prepandemic period, Charlson Index score, race, age, gender, and county average SVI as the independent variables.

Finally, we ran a regression model on the number of telehealth visits. Since only one-third of patients have a telehealth visit in their data, we fit a zero-inflated negative binomial regression model [27]. The zero-inflated model is a 2-step regression model, and in the first step a logistic regression model is fit to explain which patients have no telehealth appointment. In the second step, for the patients who have a telehealth appointment, a negative binomial regression model is fit (to best fit the distribution in the number of telehealth visits). We use the same independent variables: in-person visits in the prepandemic period, Charlson Index score, race, age, gender, and county average SVI. We finally highlight the adoption patterns of telehealth, broken down by race and SVI.

### Ethical Considerations

The original data collection and the study protocol were reviewed by the Institutional Review Board of Georgetown-Howard Universities Center for Clinical and Translational Science (approval ID: STUDY00003813). The analysis followed the approved protocol and the authors therefore had the permission to use the data for this study. The data were deidentified and the study was an except retrospective study; therefore, consent was not sought and no compensation was provided.

## Results

### Cohort Characteristics

Table 1 summarizes the cohort characteristics for the different disease groups. The average age is lowest for the anxiety and depression cohorts and highest for those experiencing kidney failure and heart failure. Interestingly, while men constitute almost half of the kidney failure and heart failure cohorts, they constitute close to 45% (15,922/35,460 and 36,930/81,885, respectively) of patients with cancer and diabetes and close to 30% (19,733/67,578 and 13,808/45,570, respectively) of those with anxiety and depression. We also observe variation by race: White patients are the majority in the anxiety, depression, and cancer cohorts, while Black patients are the majority in the diabetes, kidney failure, and heart failure cohorts. These differences reflect both expected variation by disease (ie, typically older patients have kidney and heart failure) and the demographics served by the health care system we study. Across disease cohorts, between 22% (14,597/67,578 for anxiety) and 33% (27,104/81,885 for diabetes) of patients live in counties with high SVI. The highest percentage of patients living in high-SVI counties are patients in the diabetes, kidney failure, and heart failure cohorts. Between 24% (6940/29,284 for kidney failure and 5055/21,152 for heart failure) and 32% (11,117/35,460 for cancer) of patients live in counties with low SVI.

**Table 1:** Summary statistics for the 6 disease cohorts.

			Anxiety		Depression	Diabetes		Kidney failure	Heart failure		Cancer
Cohort size, n			67,578		45,570	81,885		29,284	21,152		35,460
Age (years), mean			51.9		54.4	65.5		70.9	71.3		68.2
Charlson Index^a^			1.00		1.28	2.37		2.63	3.54		3.48
**Sex, %**											
	Female	70.7		69.7		54.9	48.9		50.3	55.1	
	Male	29.2		30.3		45.1	51.1		49.7	44.9	
	Other	0.01		0.01		0.004	—^b^		—	—	
**Race, %**											
	Black	30.3		35.0		53.4	58.2		54.3	35.3	
	Other	8.1		7.3		9.0	6.8		4.9	9.2	
	White	61.6		57.6		37.6	35.1		40.8	55.5	
**SVI^c^, %**											
	High	21.6		24.4		33.1	32.2		32.6	22.1	
	Medium	46.2		47.0		41.0	43.4		42.7	45.8	
	Low	31.3		27.9		25.2	23.7		23.9	31.5	
	Unknown	0.8		0.7		0.7	0.7		0.8	0.7	
**Number of visits^d^**											
	Pre–COVID-19	4.3		4.7		4.5	5.9		6.2	5.4	
	During COVID-19	3.8		4.0		4.0	4.8		4.8	4.3	
Any telehealth (%)^e^			35.0		33.4	30.3		31.1	27.7		32.1

^a^Without age.

^b^Not applicable.

^c^SVI: Social Vulnerability Index.

^d^Average number of visits in a 6-month period.

^e^In the pandemic period.

### Overall Usage

**Figure 1:**
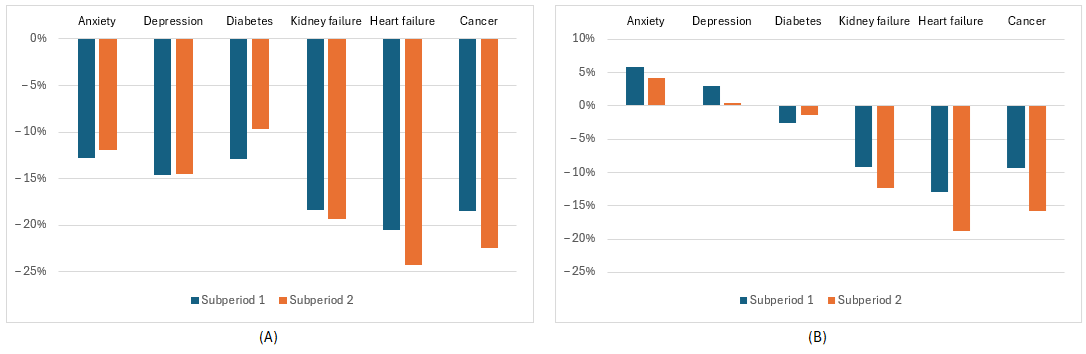
The percent difference in in-person visits (A) and overall utilization (B) during the 12-month pandemic period from June 2020 through November 2020 (subperiod 1) and December 2021 to May 2021 (subperiod 2) for the 6 disease cohorts.

Figure 1A summarizes the in-person health care usage for the different disease cohorts. In order to capture temporal patterns, we break the 12-month pandemic period into two 6-month subperiods (with June 2020 through November 2020 as pandemic subperiod 1 and December 2020 through May 2021 as pandemic subperiod 2). We note that in-person usage decreased from the prepandemic period for every disease cohort. The reduction was the greatest for cancer (–18% and –22% in pandemic subperiods 1 and 2, respectively), heart failure (–20% and –24%), and kidney failure (–18% and –19%). Smaller decreases are seen for anxiety, depression, and diabetes. We then compare Figure 1A with Figure 1B, which shows the overall usage. We observe that during the pandemic period, the number of telehealth visits exceeds the reduction in in-person visits for both depression and anxiety; we see an increase in overall usage for these disease cohorts during this period. We also note that for the diabetes cohort, the number of telehealth visits almost equals the decrease in in-person visits. However, for the cancer, heart failure, and kidney failure cohorts, there remains a significant reduction in the total number of visits.

### In-Person Visits

We run a linear regression model for each disease group for the number of visits in the pandemic period. Recall that the dependent variable was the natural logarithm of the number of in-person visits; therefore, we exponentiate the regression coefficients and interpret the coefficients as multiplicative.

The regression results are summarized in Table 2. We note that across disease groups, the higher the number of preperiod visits, the higher the expected number of pandemic period visits. We then observe that both White patients and, to a larger extent, those of other races have on average fewer in-person encounters than Black patients, after accounting for all other variables in the model. (Compared with similar Black patients, White patients have 1.8%-9.8% fewer visits and those of other races had 14.2%-19.7% fewer visits). For all diseases except heart failure, the Charlson Index is negatively associated with number of visits, indicating that, all else being equal, sicker patients have fewer visits. While the impact of age is statistically significant across diseases, the directionality of the impact differs by disease. Age is negatively associated with the number of visits for patients with both heart and kidney failure, has a small association with the number of visits for patients with diabetes and those with cancer, and is positively associated with the number of visits for anxiety and depression. Across diseases, men have on average fewer visits than women (holding everything else constant, estimates range from –5.6% for heart failure to –13.9% and –15.8% for depression and anxiety, respectively). Finally, across most diseases and holding everything else constant, patients from high and medium SVI counties have more visits on average than patients from counties with the lowest SVI (The exception are patients from high-SVI counties in the anxiety cohort, where the association is not statistically significant.)

**Table 2:** In-person regression results by disease cohort; for each disease cohort the dependent variable is the natural logarithm of the number of in-person visits during the 12-month pandemic period, June 2020 to May 2021 (*P* values are reported within parentheses).

		Anxiety, *r* (*P* value)	Depression, *r* (*P* value)	Diabetes, *r* (*P* value)	Kidney failure, *r* (*P* value)	Heart failure, *r* (*P* value)	Cancer, *r* (*P* value)
Intercept		1.12 (<.001)	1.14 (<.001)	1.37 (<.001)	1.78 (<.001)	1.84 (<.001)	1.38 (<.001)
Pre–COVID-19 visits		0.08 (<.001)	0.08 (<.001)	0.08 (<.001)	0.06 (<.001)	0.07 (<.001)	0.07 (<.001)
Charlson Index		–0.01 (<.001)	–0.02 (<.001)	–0.02 (<.001)	–0.03 (<.001)	–0.03 (<.001)	–0.03 (<.001)
Age (years)		0.005 (<.001)	0.004 (<.001)	0.0004 (.14)	–0.004 (<.001)	–0.01 (<.001)	0.001 (.002)
**Sex**							
	Male	–0.16 (<.001)	–0.14 (<.001)	–0.10 (<.001)	–0.08 (<.001)	–0.06 (<.001)	–0.10 (<.001)
	Unknown	0.27 (.47)	0.05 (.89)	–0.34 (.52)	—^a^	—	—
**Race**							
	Other	–0.15 (<.001)	–0.17 (<.001)	–0.16 (<.001)	–0.16 (<.001)	–0.17 (<.001)	–0.22 (<.001)
	White	–0.07 (<.001)	–0.07 (<.001)	–0.03 (<.001)	–0.05 (<.001)	–0.02 (.30)	–0.1 (<.001)
**SVI^b^**							
	High	–0.002 (.82)	0.02 (.09)	0.03 (.004)	0.02 (.16)	0.01 (.49)	0.07 (<.001)
	Medium	0.05 (<.001)	0.07 (<.001)	0.1 (<.001)	0.09 (<.001)	0.1 (<.001)	0.13 (<.001)
	Missing	–0.01 (.88)	–0.05 (.31)	–0.09 (.03)	–0.05 (.52)	–0.01 (.95)	–0.02 (.77)

^a^Not applicable.

^b^SVI: Social Vulnerability Index.

### Use of Telehealth

**Figure 2:**
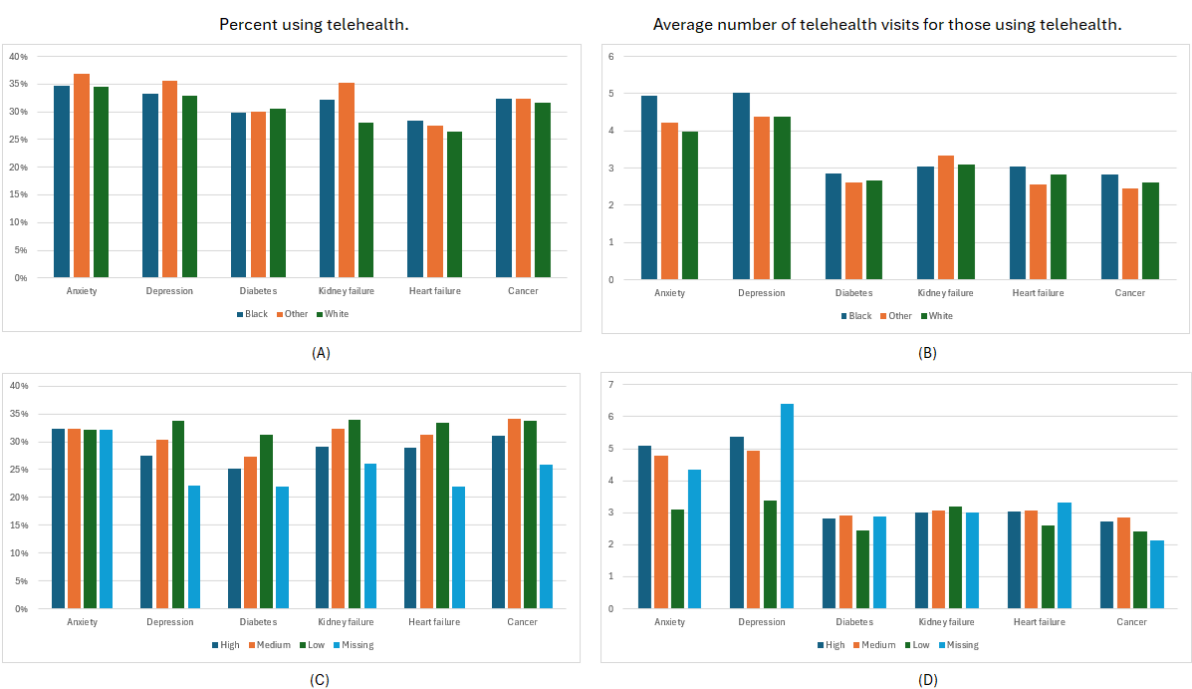
The percentage of patients who have at least 1 telehealth visit (A) and the average number of telehealth visits for patients with at least 1 visit (B), during the 12-month pandemic period broken down by race; the percentage of patients who have at least 1 telehealth visit (C), and the average number of telehealth visits for patients with at least 1 visit (D) during the 12-month pandemic period, broken down by Social Vulnerability Index group.

We studied 2 aspects of telehealth usage: first the percentage of patients using telehealth, and second the average number of telehealth visits for those patients who have at least 1 telehealth visit. In Figure 2, we break down the usage by race and SVI.

The highest rate of telehealth adoption (any usage) is by the anxiety and depression cohorts. We note that patients of other races are most likely to have at least 1 telehealth visit for both conditions (Figure 2A); however, among patients who use telehealth, Black patients have a higher average number of visits (Figure 2B). For kidney failure, heart failure, and cancer, while the rate of any telehealth usage by White patients is moderately lower than for Black patients, the average number of visits for those patients who do use telehealth are similar.

When telehealth usage for anxiety and depression is broken down by SVI, we note a more nuanced pattern. SVI does not appear to be associated with whether or not members in the Anxiety cohort use telehealth (Figure 2C), but among those who do, those with the lowest SVI have the lowest number of visits (Figure 2D). For depression we also note that patients from low-SVI areas have significantly fewer telehealth visits on average. Across all disease cohorts except anxiety, patients from low-SVI areas are more likely to use telehealth, followed by patients from medium and then high-SVI areas (Figure 2C). However, except for anxiety and depression the differences in number of telehealth visits for patients who use telehealth are generally less.

To quantify these relationships, and account for the many other factors that may affect usage, we next regress the number of telehealth visits in the pandemic period on the independent variables. Table 3 summarizes the results by disease group. For each disease group there are 2 regression coefficients. The first corresponds to the logistic regression model that explains which members do not have a telehealth visit. The second coefficient is associated with the negative binomial regression model that explains the number of visits.

We note that the more in-person pre–COVID-19 visits patients had, the less likely they are to have a telehealth visit (holding everything else constant). On average, for each additional in-person visit in the pre–COVID-19 period, the odds of not having a telehealth visit increases by 31.0% and 29.7% for anxiety and depression, respectively. We further note that across diseases, for those using telehealth, a higher number of pre–COVID-19 in-person visits is associated with fewer telehealth visits during the pandemic period.

Similarly, we note that the higher the Charlson Index, the less likely the patient is to use telehealth during COVID-19 period. However, while for kidney failure and heart failure, a higher Charlson Index is associated with a higher number of telehealth visits, for other disease groups, it is negatively associated with the number of visits.

Across disease groups, older patients are more likely to use telehealth, and for patients who do use telehealth, older patients typically have a higher number of visits (holding everything else constant). Male patients are similarly more likely to use telehealth and have a higher number of telehealth than female patients (holding everything else constant).

For anxiety, Black patients are less likely to use telehealth than White patients, and for diabetes, kidney failure, and heart failure this is reversed: Black patients in these cohorts are more likely to have at least 1 telehealth appointment than White patients (holding everything else constant). Across diseases, among those who use telehealth, White patients have a higher number of visits on average, holding everything else constant.

Finally, there is a diverging relationship between SVI group and telehealth usage. Patients from high-SVI counties are less likely to use telehealth across all disease cohorts (except for kidney failure). However, among patients who do use telehealth, patients from high-SVI counties have a higher number of telehealth visits on average than patients from low-SVI counties (everything else being the same).

**Table 3:** Telehealth regression results for the 6 disease cohorts: for each disease group a zero-inflated negative binomial regression model is run; the first column corresponds to a logistic regression model that explains which members do not have a telehealth visit in the 12-month pandemic period; and the second column reflects the results from a negative binomial regression model that explains the number of visits in the same period (*P* values are reported within parentheses below each regression coefficient).

				Anxiety, *r* (*P* value)					Depression, *r* (*P* value)					Diabetes, *r* (*P* value)					Kidney failure, *r* (*P* value)				Heart failure, *r* (*P* value)					Cancer, *r* (*P* value)		
Variable				No visits		How many		No visits			How many		No visits			How many		No visits			How many	No visits			No visits		No visits			How many
Intercept				1.9 (<.001)		0.7 (<.001)		1.96 (<.001)			0.79 (<.001)		1.51 (<–.001)			0.15 (<.001)		1.84 (<.001)			–0.48 (<.001)	1.34 (<.001)			–0.25 (<.001)		0.85 (<.001)			–0.29 (<.001)
Pre–COVID-19 visits				0.27 (<.001)		–2.28 (<.001)		0.26 (<.001)			–2.33 (<.001)		0.17 (<.001)			–2.43 (<.001)		0.14 (<.001)			–2.28 (<.001)	0.15 (<.001)			–2.67 (<.001)		0.15 (<.001)			–1.73 (<.001)
Charlson Index				0.03 (<.001)		–0.02 (<.001)		0.0003 (<.001)			–0.01 (<.001)		0.09 (<.001)			–0.001 (<.001)		0.06 (<.001)			0.05 (<.001)	0.07 (<.001)			0.02 (<.001)		0.11 (<.001)			–0.01 (<.001)
Age (years)				–0.01 (<.001)		0.0004 (<.001)		–0.01 (<.001)			–0.001 (<.001)		–0.01 (<.001)			0.01 (<.001)		–0.01 (<.001)			0.02 (<.001)	–0.01 (<.001)			0.02 (<.001)		–0.01 (<.001)			0.01 (<.001)
**Sex**																														
	Male		–0.002 (<.001)		0.26 (<.001)		0.001 (<.001)			0.3 (<.001)		–0.2 (<.001)			0.12 (<.001)		–0.11 (<.001)			0.1 (<.001)		–0.16 (<.001)		0.1 (<.001)		–0.07 (<.001)			0.09 (<.001)	
	Unknown		–1.48 (<.001)		–1.86 (<.001)		–1.27 (<.001)			–5.12 (<.001)		–0.72 (<.001)			–0.17 (<.001)		—^a^			—		—		—		—			—	
**Race**																														
	Other		–0.1 (<.001)		–0.09 (<.001)		–0.09 (<.001)			–0.09 (<.001)		–0.06 (<.001)			0.03 (<.001)		0.12 (<.001)			0.01 (<.001)		–0.14 (<.001)		0.01 (<.001)		–0.16 (<.001)			0.01 (<.001)	
	White		–0.09 (<.001)		0.03 (<.001)		0.01 (<.001)			0.05 (<.001)		0.03 (<.001)			0.02 (<.001)		0.12 (<.001)			(<.001)		0.07 (<.001)		0.13 (<.001)		–0.003 (<.001)			0.02 (<.001)	
**SVI^b^**																														
		Low	–0.47 (<.001)		–0.29 (<.001)		–0.47 (<.001)			–0.23 (<.001)		–0.14 (<.001)			–0.41 (<.001)		0.07 (<.001)			–0.3 (<.001)		–0.12 (<.001)		–0.5 (<.001)		–0.08 (<.001)			–0.07 (<.001)	
		Medium	–0.004 (<.001)		–0.14 (<.001)		–0.06 (<.001)			–0.17 (<.001)		0.05 (<.001)			–0.16 (<.001)		0.05 (<.001)			–0.19 (<.001)		0.03 (<.001)		–0.19 (<.001)		0.11 (<.001)			–0.27 (<.001)	
		Missing	–0.1 (<.001)		0.04 (<.001)		0.22 (<.001)			0.23 (<.001)		0.07 (<.001)			0.29 (<.001)		–0.002 (<.001)			0.33 (<.001)		0.2 (<.001)		0.14 (<.001)		–0.31 (<.001)			–0.01 (<.001)	

^a^Not applicable.

^b^SVI: Social Vulnerability Index.

## Discussion

### Principal Findings

This study compares the impact of COVID-19 pandemic on health care usage across multiple disease cohorts. We found that, as reported in previous studies, in-person visits decreased across all disease cohorts. The largest decreases in in-person visits were observed among patients with cancer, heart failure, and kidney failure. There are a number of possible reasons for this, including fear of COVID-19 infection, as all of these diseases are risk factors for COVID-19 complications, and age, as the average age was also the highest in these cohorts [28]. Across diseases, we found that the higher the pre–COVID-19 health care usage, the higher the in-person usage in the pandemic period, and the lower the odds of using telehealth. Interestingly, conditioned on a person using telehealth, the higher the pre–COVID-19 usage, the lower the number of pandemic period telehealth appointments, perhaps indicating that sicker patients were being routed to in-person visits.

Telehealth usage for some disease cohorts was equivalent to the reduction in in-person visits. In the 2 mental health cohorts we studied, the number of telehealth visits exceeded the reduction of in-person visits, leading to an increase in overall health care usage for both anxiety and depression. In contrast to the other diseases we studied, depression and anxiety may be treated and managed without physical examination [29,30], and therefore these findings are not surprising. Our finding that telehealth adoption is concurrent with the decrease in in-person mental health appointments is congruent with other recent studies [31,32]. There are additional possible explanations for the increase in usage among the anxiety and depression cohorts, including the impact of the COVID-19 pandemic on mental health.

During the pandemic, telehealth provided access to care and may have supplemented in-person visits to varying degrees. Therefore, it is critical that this mode of health care delivery does not introduce new disparities or exacerbate preexisting ones. When studying the impact of patients’ demographic characteristics association with telehealth usage, we find that women (except for anxiety and depression) and younger patients were less likely to use telehealth, consistent with previous publications [16-19]. In contrast to earlier works, we do not find consistent differences between telehealth adoption of Black and White patients after accounting for all other covariates. However, conditioned on that patients use telehealth, and accounting for all other factors, White patients have more visits on average than Black patients. This is in contrast with the average numbers presented in Figure 2, highlighting the importance of accounting for the many factors influencing telehealth adoption with regression modeling.

The observed relationship between SVI and telehealth usage patterns offers nuanced understanding of telehealth adoption across SVI areas. Across disease groups, patients from low-SVI counties are more likely to use telehealth but for fewer visits on average. On the other hand, patients from high-SVI counties are less likely to use telehealth, but if they do, they use telehealth more on average. Understanding barriers to telehealth adoption, including inadequate connectivity, lack of privacy, and other hidden barriers, is therefore especially important as telehealth visits may become a standard of care.

Telemedicine can facilitate effective communication between patients and health care workers. However, some patients find it difficult to adapt to telehealth communication. In addition, a number of older patients who are not as familiar with telehealth communication reported a relative lack of an emotional connection with the health care worker compared with in-person visits [33]. Telehealth consultations have been found to be more effective in primary care appointments and mental health consultations than they are for patients with high-risk conditions, based on both patient feedback and staff evaluations [8]. Our results reflect previous observations in which telehealth visits did not compensate for decreased in-person visits for patients with cancer, heart failure, or kidney failure, who experienced the greatest pandemic-related decrease in visits. As patients with these diagnoses require frequent visits to ensure stabilization of these chronic conditions, further research is needed to understand these differences. Our study also indicates that sicker patients (as captured by the Charlson index, and accounting for all other covariates) are less likely to use telehealth, and if they do they have lower number of telehealth visits on average, except for patients in the heart failure and kidney failure groups. Further study is needed to understand the nuances behind these usage patterns.

### Limitations

This study has several limitations. In only rare cases did we observe death of patients in our study population, so we cannot confirm whether some of the reduction in visits is due to mortality in our disease cohorts. We note that cancer, heart failure, and kidney failure were the 3 disease cohorts with the largest reduction in visits in the second pandemic subperiod, and this may reflect higher mortality rates in these cohorts. We also note that we do not observe patient health care encounters outside the regional health system. Therefore, the extent to which patients moved away or sought services elsewhere is not captured. Finally, since our cohort has limited geographical scope, the results may not generalize beyond the regional service area.

### Conclusions

The COVID-19 pandemic has affected health care usage across multiple disease conditions. The impacts of the pandemic on health care usage and the role of telehealth in replacing in-person visits vary among different disease cohorts. Understanding these differences can inform current practices and provides opportunities to further guide modalities of in-person and telehealth visits. Critically, further study is needed to ensure that all patients have equal access to telehealth services. A better understanding of the role of social determinants of health may lead to more support for patients and help individual health care providers improve access to care for patients with chronic conditions.

In summary, this study offers a unique comparison across disease cohorts from the same health system. In doing so, we highlight that many of the previously observed demographic differences consistently hold across disease cohorts. The study further takes a detailed analytical approach to study telehealth adoption offering new insights. For example, the study highlights that while telehealth adoption is lower for high SVI areas, if patients from high SVI areas use telehealth, then their usage exceeds that of other patients from lower SVI areas.

## Appendix

Multimedia Appendix 1 Health care codes used to identify our disease cohorts, and sensitivity analysis complementing the main results of the paper.

## Abbreviations

ICD-9: International Classification of Diseases, Ninth Revision
ICD-10: International Statistical Classification of Diseases, Tenth Revision
SVI: Social Vulnerability Index

## Data Availability

The data sets generated for and analyzed during this study are not publicly available due the approved study protocol restrictions and patient privacy considerations. The data can be made available following a study protocol review and an appropriate institutional review board approval.
