# Supplementary material for: Health Care Usage During the COVID-19 Pandemic and the Adoption of Telemedicine: Retrospective Study of Chronic Disease Cohorts
Source: J Med Internet Res. 2024 Oct 3;26:e54991. doi: 10.2196/54991 (PMC11487209; doi:10.2196/54991)
Supplement: Multimedia Appendix 1 [file jmir_v26i1e54991_app1.pdf]

Multimedia Appendix 1

A. Disease identification

We study six disease cohorts: anxiety, depression, diabetes, kidney failure, heart failure, and cancer). We defined a disease cohort of patients to include any patient diagnosed with the disease using the corresponding International Classification of Diseases, Ninth Revision (ICD-9) and International Classification of Diseases, Tenth Revision (ICD-10) codes provided in Table A.1.

Table A.1: Summary of ICD-9 and ICD-10 codes used to define each disease cohort.

Disease	ICD-10 and ICD-9 codes
Anxiety	F40-F48
Depression	F33.% / 296.3%
Diabetes	E10.%, E11.% / 250.%
Kidney Failure	N18.% / 585.%
Heart Failure	I50.% / 428.%
Cancers	C% / 140.%-239.%

B. Main results for patient cohorts where each patient is in a single cohort

We rerun our main analysis, but this time we only include patients that had a single study condition. We observe that the average disease burden in each cohort has decreased, as we have removed patients with multiple study diseases. This fact is reflected in the results of the in-person regression models for the single study disease cohorts reported in Table B.1. We also note that the intercepts are all lower compared to the full cohorts, especially for the kidney failure and heart failure cohorts.

Most of the study's main observations hold. Across diseases, the higher the number of pre-pandemic visits, the higher the number of visits during the pandemic period. Across diseases, otherwise similar male patients have a lower number of visits than female patients, but the effect size is reduced for the diabetes and kidney failure cohorts, and it is small and not significant for the heart failure cohort. Also as in our main study, otherwise similar non-White, non-Black patients have fewer visits than White and Black patients, and the effect sizes are large and consistent with the main results. For anxiety, depression, diabetes and cancer, otherwise similar White patients have fewer visits than Black patients, again consistent with the main study. For the kidney failure cohort, the effect of being White is no longer statistically significant when studying the single disease cohorts, and in contrast to the main study, White patients in the heart failure cohort have more in-person visits than otherwise similar Black patients. Finally, we observe that across all disease cohorts except for anxiety, the impact of high SVI is statistically insignificant, but for many medium SVI counties the impact is statistically significant and positive (all other factors being equal); indicating higher number of visits on average compared to patients from low SVI counties.

Table B.1: In-person regression results by disease groups for single study disease cohorts.[a]

		Anxiety	Depression	Diabetes	Kidney failure	Heart failure	Cancer
	Intercept	1.01 (<0.001)	0.91 (<0.001)	1.06 (<0.001)	1.33 (<0.001)	1.38 (<0.001)	1.14 (<0.001)
	Pre-COVID visits	0.09 (<0.001)	0.08 (<0.001)	0.1 (<0.001)	0.07 (<0.001)	0.09 (<0.001)	0.08 (<0.001)
	Charlson Index	0.02 (0.11)	0.01 (0.27)	-0.01 (0.12)	-0.03 (0.04)	-0.02 (0.19)	-0.02 (<0.001)
	Age	0.004 (<0.001)	0.004 (<0.001)	0.0011 (<0.001)	-0.003 (0)	-0.01 (<0.001)	0.002 (<0.001)
Gender	Male	-0.15 (<0.001)	-0.13 (<0.001)	-0.05 (<0.001)	-0.08 (0)	-0.005 (0.87)	-0.06 (<0.001)
	Unknown	0.33 (0.42)	0.64 (0.28)	-0.07 (0.9)			
Race	Other	-0.14 (<0.001)	-0.17 (<0.001)	-0.13 (<0.001)	-0.15 (<0.001)	-0.15 (0.02)	-0.22 (<0.001)
	White	-0.04 (<0.001)	-0.06 (0.003)	-0.04 (0.002)	0.02 (0.42)	0.11 (0.001)	-0.13 (<0.001)
SVI	High	-0.055 (<0.001)	-0.003 (0.91)	0.01 (0.34)	0.03 (0.39)	-0.01 (0.72)	0.02 (0.45)
	Medium	0.01 (0.5)	0.05 (0.01)	0.08 (<0.001)	0.19 (<0.001)	0.11 (0.004)	0.12 (<0.001)
	Missing	-0.06 (0.32)	-0.2 (0.06)	-0.11 (0.06)	0.05 (0.76)	0.08 (0.67)	-0.06 (0.5)

[a] *P-values* reported in parentheses.

We finally compare the telehealth utilization results for the single study disease cohort. We note that since these disease cohorts are smaller than those in the main study, the results are less statistically significant; however, many of the main effects hold.

Consistent with our main results, a higher number of pre-COVID visits is associated with lower odds of utilizing telehealth during the pandemic. Similarly, and again consistent with our main results, a higher number of pre-COVID visits is associated with lower number of telehealth visits during the pandemic. For anxiety, depression and heart failure, being male is negatively associated with both utilizing telehealth and but positively with the number of visits (i.e. conditioned on using telehealth men in these disease cohorts use telehealth more than women, holding everything else constant). In contrast, being male is positively associated with utilizing telehealth in the diabetic, kidney failure and cancer cohorts.

Overall, being Black (compared to White) is negatively associated with utilizing telehealth across disease cohorts (except diabetes, where the impact is not statistically significant), and being Black is further associated with higher number of telehealth visits than being White, which is in contrast with the results of the main study. Finally, for anxiety, depression, and diabetes, living in a high SVI county is associated with lower likelihood of utilizing telehealth and a higher number of telehealth visits, compared to being from a low SVI county, again consistent with our main study.

Table B.2: Telehealth regression results by disease groups.[a]

		Anxiety		Depression		Diabetes		Kidney failure		Heart failure		Cancer	
	Variable	No visits	How many	No visits	How many	No visits	How many	No visits	How many	No visits	How many	No visits	How many
	Intercept	1.29 (<0.001)	1.22 (<0.001)	0.73 (<0.001)	1.64 (<0.001)	0.76 (<0.001)	0.41 (<0.001)	1.87 (<0.001)	-0.2 (0.22)	-0.004 (0.98)	-0.62 (0.04)	-0.6 (<0.001)	-0.62 (<0.001)
	Pre-COVID visits	1.05 (<0.001)	-1.76 (<0.001)	1.03 (<0.001)	-1.05 (<0.001)	0.35 (<0.001)	-2.08 (<0.001)	0.15 (0.54)	-1.93 (0.02)	0.31 (0.30)	-1.9 (0.03)	0.04 (0.60)	-1.63 (0.002)
	Charlson Index	0.08 (<0.001)	-0.05 (0.04)	0.009 (0.72)	-0.02 (0.63)	0.1 (<0.001)	0.04 (0.04)	0.04 (0.05)	0.04 (0.33)	0.07 (0.02)	0.14 (0.004)	0.16 (<0.001)	0.03 (0.02)
	Age	-0.01 (<0.001)	-0.0038 (<0.001)	-0.003 (0.005)	-0.009 (<0.001)	-0.01 (<0.001)	0.01 (<0.001)	-0.02 (<0.001)	0.01 (<0.001)	0.01 (0.01)	0.02 (<0.001)	0.01 (<0.001)	0.02 (<0.001)
Gender	Male	0.045 (0.01)	0.21 (<0.001)	0.188 (<0.001)	0.28 (<0.001)	-0.16 (<0.001)	0.05 (0.19)	-0.15 (<0.001)	0.09 (0.19)	0.11 (0.11)	0.18 (0.09)	-0.13 (<0.001)	-0.08 (0.11)
	Unknown	-2.52 (0.01)	-11.02 (0.98)	0.15 (0.85)	-1.2 (0.47)	-0.14 (0.88)	-1.02 (0.64)						
Race	Other	0.05 (0.04)	-0.17 (0.002)	-0.08 (0.24)	-0.12 (0.25)	0.03 (0.44)	0.08 (0.14)	0.12 (0.05)	-0.09 (0.44)	-0.37 (0.05)	-0.03 (0.90)	-0.2 (0.001)	-0.1 (0.29)
	White	-0.05 (0.01)	-0.18 (<0.001)	-0.06 (0.14)	-0.17 (0.01)	0.01 (0.75)	-0.18 (<0.001)	-0.1 (0.06)	-0.01 (0.94)	-0.25 (0.001)	-0.5 (<0.001)	-0.145 (<0.001)	-0.17 (0.01)
SVI	Low	-0.37 (<0.001)	-0.46 (<0.001)	-0.17 (0.003)	-0.54 (<0.001)	-0.08 (0.01)	-0.49 (<0.001)	0.23 (<0.001)	-0.03 (0.72)	-0.07 (0.44)	-0.6 (<0.001)	0.03 (0.56)	-0.05 (0.19)
	Medium	0.055 (0.01)	-0.14 (<0.001)	0.04 (0.47)	-0.25 (<0.001)	0.02 (0.50)	-0.1 (0.02)	-0.02 (0.62)	-0.27 (0.003)	-0.33 (<0.001)	-0.2 (0.14)	0.15 (0.002)	-12.03 (0.49)
	Missing	-0.34 (<0.001)	-0.4 (0.02)	-0.01 (0.97)	0.58 (0.16)	0.06 (0.71)	0.3 (0.15)	-0.595 (0.15)	0.26 (0.67)	0.93 (<0.001)	0.13 (0.8)	-1.18 (<0.001)	0.03 (<0.001)

[a] *P-values* reported in parentheses.
